# Unraveling the stride: exploring the influence of neurogenic orthostatic hypotension on gait and balance in Parkinson’s disease

**DOI:** 10.1007/s10286-024-01071-y

**Published:** 2024-10-02

**Authors:** Gabriele Imbalzano, Claudia Ledda, Marta Maria Tangari, Carlo Alberto Artusi, Elisa Montanaro, Mario Giorgio Rizzone, Maurizio Zibetti, Leonardo Lopiano, Alberto Romagnolo

**Affiliations:** 1 Department of Neuroscience “Rita Levi Montalcini”, University of Torino, Via Cherasco 15, Turin, 10126 Italy; 2 SC Neurologia 2U, AOU Città della Salute e della Scienza, Turin, Italy; 3 Clinical Psychology Unit, AOU Città della Salute e della Scienza, Turin, Italy

**Keywords:** Parkinson’s disease, Neurogenic orthostatic hypotension, Gait, Balance, Falls

## Abstract

**Purpose:**

Neurogenic orthostatic hypotension (nOH) and gait impairment are frequent sources of disability in Parkinson’s disease (PD). However, the impact of nOH on balance and gait features remains unclear. This cross-sectional study aimed to assess the influence of nOH on postural and gait parameters in a cohort of patients with PD by means of wearable inertial sensors.

**Methods:**

Gait and balance were assessed using Opal inertial sensors. nOH was defined as sustained systolic blood pressure (BP) drop ≥ 20 mmHg or diastolic BP drop ≥ 10 mmHg within 3 min of standing, with a ΔHR/ΔSBP ratio ≤ 0.5 bpm/mmHg. Analysis of covariance was performed to evaluate differences in gait/balance features between patients with and without nOH, adjusting for age, cognitive status, and motor disability. Moreover, we performed the same analysis considering the presence of hemodynamically relevant nOH (orthostatic mean BP ≤ 75 mmHg).

**Results:**

A total of 82 patients were enrolled, 26 with nOH (31.7%), of which 13 presented with hemodynamically relevant nOH. After correcting for confounders, nOH was independently associated with lower gait speed (*p* = 0.027), shorter stride length (*p* = 0.033), longer time for postural transitions (*p* = 0.004), and increased postural sway (*p* = 0.019). These differences were even more pronounced in patients with hemodynamically relevant nOH. Higher postural sway was associated with a 7.9-fold higher odds of falls (*p* = 0.040).

**Conclusions:**

Our study presents an objective demonstration of the independent negative impact of nOH on gait and balance in PD, emphasizing the need for careful detection and management of nOH to mitigate gait and balance disturbances in PD.

## Introduction

Orthostatic hypotension (OH) and gait impairment are common sources of disability in Parkinson’s disease (PD) [1, 2]. OH in PD is mostly neurogenic (nOH), i.e., primarily caused by cardiovascular autonomic dysfunction with post-ganglionic noradrenergic denervation of heart and blood vessels [3]. nOH prevalence increases with disease progression [4], although its severity is not associated with the duration of the disease [5]. Conversely, gait impairment in PD typically worsens over time, with progressive reduction of gait speed, step length, and impaired rhythmicity, as well as the onset of episodic gait impairment, such as freezing of gait (FoG) [6, 7]. Several studies have shown a close relationship between OH and faster motor and cognitive impairment [2, 8, 9], which may eventually result in increased risk of falls [10–12], postural instability [13], and higher healthcare costs [14]. Moreover, an association between OH, falls, and ambulatory capacity impairment in PD was observed also in patients asymptomatic for postural lightheadedness [15].

The evaluation of kinematic measures with small inertial sensors provided reliable insights to understand the complex pathophysiology of gait and postural instability in PD [16], and it has been recently applied in the assessment of OH-related balance and gait disturbances [17, 18]. However, though showing a good accuracy in predicting OH-related falls [17], and suggesting a negative impact of OH on some gait parameters [18], these preliminary findings are burdened by small sample size and lack of a control group. Whether OH directly contributes to worse motor and cognitive performances (e.g., hemodynamic instability with transient cerebral hypoperfusion) or is merely a feature of a more malignant PD phenotype [8, 9, 19, 20], remains a matter of debate.

In this context, we aimed to explore the influence of nOH on postural and spatiotemporal gait parameters in a large sample of patients with PD, using wearable inertial sensors and correcting for motor and cognitive disability. As a secondary aim, we further evaluated whether the presence of hemodynamically relevant hypotension could determine a more pronounced effect on these parameters. Finally, as an ancillary aim, we evaluated whether falls, one of the main complications of nOH, were associated with alterations in balance or gait parameters.

## Methods

We screened 95 consecutive advanced patients with PD (i.e., patients with fluctuations and dyskinesia that can no longer be satisfactorily treated with oral medication [21]) who were candidates for device-aided therapies (deep brain stimulation or levodopa carbidopa intestinal gel) at the Movement Disorder Center of the Turin University Hospital. The study was conducted between October 2021 and January 2023. The local institutional review board approved the study, and all participants gave written informed consent. The study was conducted in accordance with the ethical standards laid down in the 1964 Declaration of Helsinki and its later amendments.

### Eligibility criteria

Inclusion criteria were: a diagnosis of idiopathic PD, as per the Movement Disorders Society (MDS) criteria [22]; a stable dose of dopaminergic therapy and antihypotensive or antihypertensive medications for at least 4 weeks; and a Hoehn and Yahr (H&Y) score ≤ 4 in OFF therapeutic condition [23]. Exclusion criteria were: neurological signs suggestive of a diagnosis other than idiopathic PD; diabetes mellitus or other condition associated with autonomic neuropathy; cardiac arrhythmia, diagnosis of cardiac valve disease, or clinically relevant cardiac structural abnormalities; treatment with alpha-adrenergic antagonists for prostatic disorders; orthopedic or arthritic conditions and/or previous orthopedic surgeries severe enough to influence balance and gait parameters; and inability to walk independently for 10 m and for at least 60 s in OFF therapeutic condition.

### Patient assessment

Clinical and demographic data included age, gender, medical history, duration of PD, medication regimen, PD symptoms and their fluctuations as per the MDS-Unified Parkinson’s Disease Rating Scale (MDS-UPDRS) Parts I–IV, occurrence of falls during the previous month, and levodopa equivalent daily dose (LEDD) calculated according to a validated conversion table [24]. The Montreal Cognitive Assessment (MoCA) was used for general cognitive assessment [25]. When available, we also collected genetics data.

Patients underwent blood pressure (BP) measurements using an automated sphygmomanometer (HEM-7200-Omron Healthcare Co. Kyoto, Japan) placed at heart level on the left arm, in the following conditions: (a) after a minimum of 10 min of supine rest; (b) after 1 min and 3 min of active standing. To minimize BP variability due to antiparkinsonian medications [26], BP assessments were performed during the practically defined OFF condition (following an overnight withdrawal of antiparkinsonian medications), at least 3 h after the last meal. Moreover, patients were told to avoid taking their usual vasoactive therapy in the previous 12 h. OH was defined as a BP drop ≥ 20 mmHg systolic or 10 mmHg diastolic within 3 min of active standing [27]. Patients with a rise in heart rate (HR)/fall in systolic BP ratio > 0.5 beats per minute (bpm)/mmHg (ΔHR/ΔSBP ratio) were considered as having non-neurogenic OH [28] and were excluded from the analyses. Hemodynamically relevant nOH was defined as orthostatic mean arterial pressure (MAP) ≤ 75 mmHg [29].

### Gait and balance analysis

Within 5 min of the BP measurements, all patients underwent instrumental evaluation of gait and balance parameters using wearable inertial sensors (Opal, APDM’s Mobility Lab system) placed at multiple points in the upper and lower body (two sensors attached on the feet, two at outer surface of the thighs, one at right hip joint, one on the sternum, and one at lumbar level) [30]. The Opal sensor includes a triaxial accelerometer, gyroscope, and magnetometer with a sampling rate of 128 Hz. We collected gait and balance outcomes, directly processed from the manufacturer-provided software with validated algorithm [31]. The tasks consisted of a battery of standardized motion tests, conducted in a dedicated room:

• Sway test, with patients asked to remain in balance during an upright standing position for 30 s, with arms at rest and eyes closed. This is a test for evaluation of quiet stance balance. The parameter considered was the root mean square (RMS) area (m/s^2^), RMS of the sway angle in both the coronal and sagittal planes.

• 360° Turn Test, with patients required to start from standing position, make a turn 360° clockwise, and as soon as they can to return to the initial position, 360° counterclockwise. This is a measure of dynamic balance. The parameters considered were the turn duration (s) and the turn velocity (degree/s).

• Timed Up and Go (TUG) test, with subjects asked to stand up from a chair, walk 3 m straight, turn around, walk back, and sit down, at a comfortable pace. This is a test for measure of postural transitions. We considered the following outputs: the TUG duration (s), the turn duration (s), the turn velocity (degree/s), and the sit to stand duration (s).

• 2 min walking test (2MWT): this test was conducted in adequate space to allow a 2 min walk, back and forth in a straight line and performing tight turnings, at a comfortable pace and with a distance of 10 m per lap. This test measures full body gait, asymmetry, variability, and turning. The outputs considered were: gait speed (m/s), double support [the percentage of the gait cycle time (GCT) in which both feet are on the ground], stride length (m), and step duration (s). Gait speed was calculated as the forward distance covered during an entire gait cycle, divided by the gait cycle duration. The data of each gait cycle were then averaged to provide the mean value of the entire test. Data of the turning phases, computed from the angle changes and the angular velocity detected from the sternum sensor, were automatically excluded from the speed analysis to improve its sensitivity.

As for BP measurement, gait and balance evaluations were also performed in the practically defined OFF condition.

### Statistical analysis

Clinical and demographic characteristics are reported as mean ± standard deviation and range, or absolute number and percentages, as appropriate. Differences between patients with and without nOH (nOH^+^ and nOH^−^) were evaluated by means of the Mann–Whitney nonparametric test or Fisher’s exact test, as appropriate.

As primary outcome, the analysis of covariance (ANCOVA) was conducted to evaluate differences in gait and balance parameters between the two groups, corrected for patient’s age, cognitive status evaluated with MoCA, motor symptoms severity evaluated with MDS-UPDRS III total score, and postural instability and gait difficulty (PIGD) score (sum of MDS-UPDRS items 3.9, 3.10, 3.12, 3.13; i.e., arising from chair, gait, postural stability, posture) [32] (covariates). Moreover, a linear regression analysis was applied to evaluate the correlation between BP changes and gait or postural parameters, using SBP drop as independent variable and correcting for age, MoCA score, MDS-UPDRS III total score, and PIGD score.

As secondary outcome, a new ANCOVA analysis with the same covariates was conducted to evaluate differences among patients without OH, patients with non-hemodynamically relevant nOH (i.e., orthostatic MAP > 75 mmHg), and patients with hemodynamically relevant nOH (i.e., MAP ≤ 75 mmHg). To evaluate the association between nOH (and hemodynamically relevant nOH) and falls, we used a binary logistic regression analysis, adjusting for age, MoCA, MDS-UPDRS part III, and PIGD scores. Then, a second binary logistic regression analysis was conducted to evaluate the association between falls and each gait and balance parameter, adjusting for the same confounders and for the presence of nOH. Bonferroni adjustment for multiple comparisons was applied to conduct post hoc pairwise analyses, and ANCOVA assumption of homogeneity of regression slopes was verified. All analyses were performed by Statistical Package for the Social Sciences (SPSS 27.0 for Macintosh, Chicago, IL), using two-tailed *p*-values with a level of significance of 0.05.

## Results

Out of 95 PD patients screened during the study period, 4 (two with and two without nOH) were not able to complete the balance and gait trials in the medication OFF condition due to their severe axial symptoms, and 5 presented with diabetes mellitus. After the first screening, we also excluded four patients with non-neurogenic OH. Thus, a total of 82 consecutive patients (61 male and 21 female) were enrolled in the study: 26 patients presented with nOH (nOH^+^, 31.7%), with 13 of them (50%) meeting the criteria for hemodynamically relevant nOH. All the 82 patients completed the full instrumented assessment. Patients with nOH were older (63.5 ± 6.8 versus 59.6 ± 7.0 years, *p* = 0.009), and had lower MoCA score (22.4 ± 2.9 versus 24.4 ± 2.9, *p* = 0.004). Patients presented similar LEDD (1208.1 ± 416.1 versus 1267.6 ± 438.4, *p* = 0.501) and disease duration (10.7 ± 3.0 versus 12.8 ± 5.7, *p* = 0.090). Patients with nOH^+^ showed also higher scores at the MDS-UPDRS part I, II, and III, and higher H&Y stage. Genetic testing was available for 25 patients (*N* = 23 in the nOH^−^ group, *N* = 2 in the nOH^+^ group), of which 3 presented with PRKN variant, 2 with GBA variant, and 2 with LRRK2 variant. None of these patients had nOH. Patients’ demographical and clinical characteristics are reported in Table 1.

**Table 1:** Patients’ demographic and clinical features

	All patients	nOH^−^	nOH^+^	*p*-Value
*N* of patients (male/female)	82 (61/21)	56 (43/13)	26 (18/8)	0.319
Age at evaluation (years)	60.9 ± 7.1	59.6 ± 7.0	63.5 ± 6.8	**0.009**
Disease duration (years)	12.2 ± 5.1	12.8 ± 5.7	10.7 ± 3.0	0.090
MoCA	23.8 ± 3.1	24.4 ± 2.9	22.4 ± 2.9	**0.004**
Total LEDD (mg)	1248.7 ± 429.8	1267.6 ± 438.4	1208.1 ± 416.1	0.501
MDS-UPDRS I	13.9 ± 5.4	12.3 ± 5.1	17.3 ± 4.3	** < 0.001**
MDS-UPDRS II	16.5 ± 7.1	14.9 ± 5.7	20.0 ± 8.4	**0.009**
MDS-UPDRS III—OFF	50.3 ± 17.1	47.2 ± 15.8	56.9 ± 18.3	**0.019**
MDS-UPDRS III—PIGD score—OFF	6.4 ± 3.8	5.8 ± 3.3	7.6 ± 4.5	0.112
Hoehn and Yahr stage—OFF	2.8 ± 0.8	2.6 ± 0.7	3.1 ± 1.0	**0.015**
MDS-UPDRS IV	10.9 ± 2.7	10.7 ± 2.8	11.3 ± 2.6	0.288
*N* of fallers in the previous month (%)	26 (31.7)	14 (25.0)	12 (46.1)	**0.023**

Bold values denote statistical significance at the *p* < 0.05 level

Results are reported as mean ± standard deviation or absolute values, as appropriate. *p*-Value is calculated comparing nOH^−^ and nOH^+^ group as per the Mann–Whitney test, or the Fisher exact test, as appropriate

*nOH*^−^ patients without neurogenic orthostatic hypotension, *nOH*^+^ patients with neurogenic orthostatic hypotension, *MoCA* Montreal Cognitive Assessment, *LEDD* levodopa equivalent daily dose, *MDS-UPDRS* Movement Disorder Society-Unified Parkinson’s Disease Rating Scale, *PIGD* postural instability gait disorder

### Comparison between patients with and without nOH

After correcting for age, cognitive status, and motor symptoms severity, patients with nOH^+^ presented lower gait speed [corrected mean ± standard error: 0.574 ± 0.042 versus 0.693 ± 0.029 m/s; F(1,5) = 5.125; *p* = 0.027], significantly correlated with SBP drop magnitude (*β* = −0.190; *R*^2^ = 0.463; *p* = 0.042), and shorter stride length (corrected mean ± standard error 0.647 ± 0.044 versus 0.765 ± 0.029 m; F(1,5) = 4.721; *p* = 0.033), without significant correlation with SBP drop (*p* = 0.217). On the contrary, step duration (*p* = 0.399) and double support percentage (*p* = 0.869) were not different between the two groups. Patients with nOH^+^ showed also a longer time to perform the sit to stand during the TUG test [corrected mean ± standard error: 1.388 ± 0.051 versus 1.198 ± 0.033 s; F(1,5) = 8.710; *p* = 0.004], significantly correlated with SBP drop (*β* = 0.449; *R*^2^ = 0.494; *p* < 0.001), while no differences were observed between the two groups during TUG for total duration (*p* = 0.407), duration of the stand to sit transition (*p* = 0.153), and for turning (turn velocity *p* = 0.415; turn duration *p* = 0.828). For balance measures, the sway test showed higher RMS sway in patients with nOH^+^ [corrected mean ± standard error: 0.979 ± 0.059 versus 0.797 ± 0.042 m/s^2^; F(1,5) = 5.796; *p* = 0.019], significantly correlated with SBP drop (*β*  = 0.349; *R*^2^ = 0.440; *p* = 0.013). The inertial measures did not show differences between the two groups at the 360° turn test. Table 2 presents the uncorrected gait and balance data of all patients.

**Table 2:** Patients’ gait and balance assessment

	All patients	nOH^−^	nOH^+^	*p*-Value
**2 min walking test**				
Gait speed (m/s) *Normative 1.04–1.64 m/s*	0.65 ± 0.26	0.70 ± 0.23	0.53 ± 0.26	**0.004**
Step duration (s) *Normative 0.45–0.58 s*	0.58 ± 0.11	0.59 ± 0.11	0.55 ± 0.12	0.187
Stride length (m) *Normative 1.11–1.66 m*	0.73 ± 0.28	0.79 ± 0.25	0.59 ± 0.29	**0.004**
Double support (%GCT) *Normative 12.4–24.6%*	27.61 ± 6.91	27.59 ± 7.50	27.66 ± 5.37	0.520
**Timed up and go**				
Duration (s) *Normative 6.28–11.6 s*	27.76 ± 25.97	27.65 ± 25.92	28.03 ± 26.74	0.661
Turn velocity (°/s) *Normative 158–322°/s*	132.23 ± 42.71	133.91 ± 41.21	128.28 ± 46.85	0.582
Turn duration (s) *Normative 1.42–2.53 s*	2.74 ± 0.56	2.73 ± 0.60	2.79 ± 0.49	0.555
Sit to stand duration (s) *Normative 0.69–1.27 s*	1.26 ± 0.24	1.18 ± 0.22	1.42 ± 0.26	** < 0.001**
**360° turn test**				
Turn duration (s) *Normative 1.86–3.80 s*	4.49 ± 2.17	4.38 ± 1.87	4.81 ± 2.90	0.870
Turn velocity (°/s) *Normative 146–359 °/s*	112.93 ± 50.01	114.23 ± 49.51	109.25 ± 52.78	0.483
**Sway test**				
RMS sway (m/s^2^) *Normative 0.231–0.661 m/s*^*2*^	0.86 ± 0.31	0.78 ± 0.29	1.01 ± 0.30	**0.002**

Bold values denote statistical significance at the *p* < 0.05 level

Results are reported as mean ± standard deviation. *p*-Value is calculated as per the Mann–Whitney test, comparing nOH^−^ and nOH^+^ group

Normative values of the mobility lab software are reported for each gait or balance evaluation conducted

*nOH*^−^ patients without neurogenic orthostatic hypotension, *nOH*^+^ patients with neurogenic orthostatic hypotension, *GCT* gait cycle time, *RMS* root mean square

### Role of Hemodynamically relevant OH

**Fig. 1:**
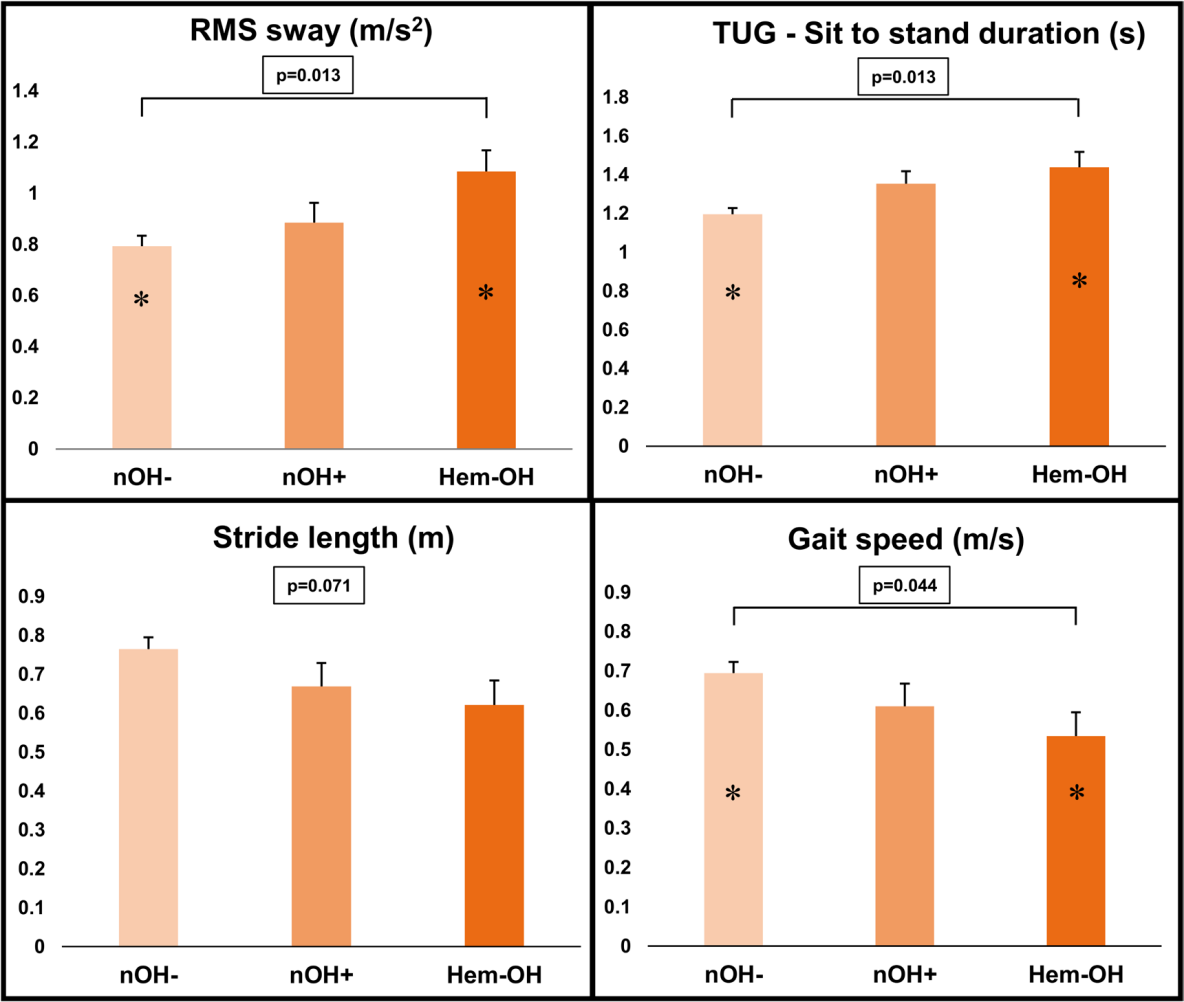
The role of hemodynamically relevant OH. The graph reports significant gait and balance parameters at the ANCOVA analysis conducted to evaluate differences between nOH^−^ patients, patients with non-hemodynamically relevant nOH (nOH^+^), and patients with hemodynamically relevant nOH (Hem-OH). Columns represent corrected mean values, and bars the standard error. *p*-Value of the test is shown for each parameter. The * indicates significant difference at post hoc pairwise analyses

When dividing the patients with nOH^+^ into non-hemodynamically and hemodynamically relevant OH, after correcting for the same covariates, the latter showed lower gait speed [F(2,6) = 3.102; *p* = 0.044], longer duration of the sit to stand transition during the TUG test [F(2,6) = 4.694; *p* = 0.013], a higher RMS sway [F(2,6) = 4.597; *p* = 0.013], and a trend toward shorter stride length, without reaching statistical threshold (*p* = 0.071) (Fig. 1). At the pairwise comparisons, after Bonferroni’s correction, we found a significant difference between nOH^−^ patients and patients with hemodynamically relevant OH for the following kinematic measures: gait speed (*p* = 0.045), sit to stand TUG duration (*p* = 0.021), and RMS sway (*p* = 0.010) (Fig. 1).

### Association between falls, nOH, and gait/balance parameters

After adjusting for age, MoCA, MDS-UPDRS part III, and PIGD scores, patients with nOH showed significantly higher odds of falling compared with those without nOH (OR 3.458; 95% CI 1.034–11.825; *p* = 0.044); this association was even stronger when considering hemodynamically relevant OH (OR 7.033; 95% CI 1.393–15.514; *p* = 0.018). Among gait and balance parameters, only RMS sway showed a strong and significant association with falls odds (OR 7.922; 95% CI 1.102–17.953; *p* = 0.040).

## Discussion

In this cross-sectional study, we analyzed the impact of nOH on gait and balance features in a cohort of patients with advanced PD. Applying the consensus-based criteria for the diagnosis of OH [27] and the ΔHR/ΔSBP ratio [28], we found that approximately one-third of our cohort presented with nOH. This group of patients showed worse motor and cognitive performances, in accordance with previous studies [2, 8, 9]. However, even after correcting for age, cognitive status, and motor symptoms severity, nOH was independently associated with lower gait speed and stride length, longer time to stand up, and worse balance performances. Moreover, when considering the subgroup of patients with hemodynamically relevant OH [29], we found an even more pronounced impact of this condition on the same kinematic measures. Finally, we observed a strong association between greater RMS sway values and higher odds of falls.

Increased postural sway, a key feature of postural instability, was already reported in patients with PD with OH, as a result of complex interaction, only partially clarified, between OH itself and balance pathophysiology [13, 17]. One of the main hypothesis postulates a common pattern of neurodegeneration leading to both OH and postural instability. In fact, the degeneration of noradrenergic neurons of the brainstem could explain both the arterial baroreflex failure [33] and the dysfunction of coeruleo-cerebellar and coeruleo-spinal pathways, involved in postural reflex regulation [34]. However, OH may directly cause postural instability through cerebral hypoperfusion of the frontal areas responsible for balance and gait [35]. The first hypothesis is consistent with previous literature data, describing dysautonomia and severe motor symptoms as distinctive features of a more malignant PD phenotype, along with rapid eye movement (REM) sleep behavior disorder and earlier development of cognitive impairment, explained by a more diffuse and faster neurodegeneration [8]. Further, the second hypothesis is part of the so-called causative hypothesis [19, 20], indicating that dysautonomia could directly contribute to a worse PD phenotype, with cortical damage induced by repeated episodes of cerebral hypoperfusion associated with increased BP variability and/or hypertensive events. However, these two different pathological mechanisms are not mutually exclusive, as already reported for the relationship between OH and cognitive impairment [20]. In fact, in our study, patients with nOH also exhibited worse motor and cognitive performances, apparently in line with the associative hypothesis. However, our findings were adjusted for the severity of motor symptoms and cognitive status, suggesting that the independent association between greater balance impairment and nOH aligns with the causative hypothesis. Furthermore, we found an even stronger association with hemodynamically relevant nOH, a validated measure of significant cerebral hypoperfusion, as well as significant correlations between higher SBP drop and lower gait speed, longer sit to stand duration during TUG, and higher postural sway. Finally, the observation of a 3.4-fold and sevenfold higher odds of falls in nOH^+^ group, and in patients with hemodynamically relevant OH, respectively, is a further confirmation of the association between nOH and falls [9–12]. Interestingly, among the gait/balance parameters evaluated, only RMS sway showed a significant and strong association with falls. Indeed, balance alterations assessed by means of wearable sensors emerged as the most accurate measures for predicting the risk of falls even in a previous prospective study [17].

Reduced gait speed with short step length are two hallmarks of parkinsonian gait [7]. The negative impact of nOH on gait parameters observed in our sample is consistent with a previous study conducted by means of a sensored walkway on a large cohort of community-dwelling, nonparkinsonian, patients [36]. Additionally, the same authors demonstrated impaired recovery of orthostatic cerebral oxygenation in elderly patients, which correlated with slower gait speed during a walking trial conducted after a prolonged supine position. This effect was especially evident between 20 s and 3 min after standing. [37]. Another small-sampled study, encompassing the use of motion sensors, evaluated 12 patients with PD with a 2MWT conducted after 3–5 min of walking, and showed a moderate to strong correlation of gait velocity and stride length with orthostatic BP changes [18]. In this context, the poorer gait performance observed in our patients with nOH, particularly in those with hemodynamically relevant nOH, underlines the primary role of nOH in determining gait alterations. Interestingly, patients in the nOH^+^ and nOH^−^ groups did not show significant differences in the TUG test duration, although this test also includes a gait evaluation. The apparent mismatch between the findings at the 2MWT and the TUG test is probably explained by their different paradigm. In fact, in the TUG test, patients must walk a very short distance, often covered in less than 30 s. Thus, from a hemodynamic perspective, it is plausible that the TUG test could be more influenced by the presence of transient OH rather than by classic OH [11]. However, the absence of continuous BP monitoring in our protocol precludes conclusive remarks on this hypothesis. Finally, it is conceivable that one of the explanations for the poorer gait performances of patients with OH^+^ at the 2MWT is represented by the progressive manifestation of fatigue, a well-known symptom of classic OH [38, 39].

There are some limitations that temper the strength of our conclusions. First, the definition of OH was restricted to BP measurements at 3 min after standing, and not based on continuous BP monitoring. This may have excluded some patients with “classic” nOH, and did not allow for the evaluation of the influence of both transient nOH and delayed nOH [11, 40]. Second, we did not consider the presence and severity of subjective symptoms of nOH; however, we decided to perform a subanalysis of patients with hemodynamically relevant nOH (MAP < 75 mmHg), a validated measure of cerebral hypoperfusion and symptomatic OH [29]. Third, the evaluation of a cohort of patients with advanced PD could limit the generalizability of our results. Fourth, the assessment of patients in their OFF condition, though justified by the intention to avoid iatrogenic confounding effects, does not fully reflect the patients’ daily life. Finally, the cross-sectional design of the study is also a limitation.

These limitations notwithstanding, our study demonstrated, by means of a thorough instrumental characterization, the independent role of nOH in determining balance and gait disturbances in patients with PD. Our findings suggest the implementation of orthostatic BP and HR measurements in clinical practice to promote a careful detection of nOH, a potentially treatable cause of falls and gait disturbances in PD. Future research with continuous blood pressure monitoring and larger multicenter cohorts are warranted to provide additional insights into the complex relationship between cardiovascular autonomic dysfunction and motor manifestations in advanced PD.

## Data Availability

The data that support the findings of this study are available in anonymized dataset from the corresponding author, upon reasonable request.
